# Resveratrol-Supported Bioenergetics Leads to Higher Productivity and Accompanying Endoplasmic Reticulum Stress in a mAb-Producing CHO Cell Line

**DOI:** 10.3390/ijms262211146

**Published:** 2025-11-18

**Authors:** Bálint Kurucz, Péter Hajdinák, András Szarka

**Affiliations:** 1 Laboratory of Biochemistry and Molecular Biology, Department of Applied Biotechnology and Food Science, Budapest University of Technology and Economics, Szent Gellért Tér 4, H-1111 Budapest, Hungary; kuruczbalint@edu.bme.hu (B.K.); hajdinak.peter@vbk.bme.hu (P.H.); 2 Biotechnology Model Laboratory, Faculty of Chemical Technology and Biotechnology, Budapest University of Technology and Economics, Szent Gellért Tér 4, H-1111 Budapest, Hungary

**Keywords:** CHO, monoclonal antibody, protein production, resveratrol, bioenergetic background, endoplasmic reticulum stress, oxidative stress

## Abstract

Increasingly unpredictable market demands and the growing market of biosimilars all facilitate lower manufacturing costs. Cell culture media additives have significant potential to improve cell-specific productivity. It has been reported that the treatment of CHO cells with resveratrol results in a reduction in viable cell density and a significant increase in cell-specific productivity. In the present study, we apply our knowledge of resveratrol gained on immortal cell lines to elucidate the details of resveratrol’s effects on mAb-producing CHO cells. In the present study, we confirm that resveratrol causes cell cycle arrest, which results in the increased protein productivity of mAb-producing cells. We demonstrate for the first time that resveratrol induces ER stress in mAb-producing CHO lines, presumably by increasing the amount of specific protein produced. It was found that ER stress did not induce oxidative stress, and cell viability could not be enhanced by apoptosis, necroptosis, or ferroptosis inhibitors. Therefore, these cell deaths may not play a role in the process. We also describe, for the first time, that resveratrol is able to increase ATP levels in mAb-producing CHO cells, thereby providing additional energy to mAb-producing CHO cells. This increased ATP synthesis is likely due to the intensification of respiration, not an increase in the number of mitochondria.

## 1. Introduction

Orthoclone OKT3, the first therapeutic monoclonal antibody, was approved for the prevention of kidney transplant rejection in 1986 [1]. In the past forty years, monoclonal antibodies (mAbs) have revolutionized the pharmaceutical industry [2]. Approximately 70% of mAbs are produced by Chinese hamster ovary (CHO) cell lines due to their robust growth and the production of antibodies characterized by glycosylation patterns that are similar to those of human antibodies [3].

It is no coincidence that the productivity of CHO cell cultures has been the focus of considerable research and development and has driven the discovery of a range of different factors. These factors include improved reactor design, enhanced feed strategies and perfusion systems, modulated temperature conditions, and improved media composition [4,5].

Cell culture media additives, small molecules of no direct nutritional value, have significant potential to improve cell-specific productivity (qp) or reduce protein aggregation [6]. The beneficial effects that different antioxidants have on the productivity of mAb-producing CHO cells has been raised. With the treatment of CHO cells with a group of flavan-3-ols, including catechin, epicatechin, epigallocatechin gallate, gallocatechin gallate, and flavone, luteolin resulted in a reduced final viable cell count and a consequent rise in cell-specific productivity without showing a detrimental effect on cell viability [7]. Similarly, α-tocopherol at low concentrations could reduce viable cell density [8], hence raising cell-specific productivity. Finally, the treatment of CHO cells with the non-flavonoid polyphenol resveratrol results in the most pronounced reduction in viable cell density with a minimal decrease in IgG synthesis and the largest increase in cell-specific productivity [4].

The effects of resveratrol on CHO cells and their specific productivity was further investigated in a detailed study. It was revealed that resveratrol inhibited CHO cell growth in a concentration-dependent manner. The effective range of resveratrol was found to be between 25 and 50 µM. CHO cells showed a steady increase in diameter for the first 48 h before starting to return to the levels shown in the untreated control. Interestingly, resveratrol affected the cell cycle differently at different concentrations. Treatment with 25 µM resveratrol induced a decline in the proportion of S-phase cells and a rise in cells in the G1/G0 and G2/M phase 24 h after inoculation. The change in the distribution of cells was temporary, but the culture took 48 h to stabilize to the proportions seen in the untreated control. However, the treatment of CHO cells with 50 µM of resveratrol induced a dramatic decline in G1/G0-phase cells and an equally dramatic rise in S-phase cells 32 h after inoculation [9]. However, the most important observation of the study was that resveratrol could slow down cell growth and increase cell-specific protein productivity without changing the characteristics of the IgG produced [9]. Recently, our research group found that resveratrol caused cell-cycle arrest in immortal mammalian cells. It decreased the proportion of the G0/G1-phase population and increased the proportion of the S-phase population significantly in concentrations of 50 µM [10].

Since these mAbs are secretory proteins [11], they can be released through the endoplasmic reticulum–Golgi secretory pathway. Since endoplasmic reticulum (ER) is responsible for the synthesis, folding, and structural maturation of most proteins [12], ER stress, a cellular response to ER overload, often occurs during the production of mAbs. Interestingly, there is no information on whether the increased productivity induced by resveratrol triggers ER stress or not.

In the present study we aim at the further characterization of the effect of resveratrol on CHO cells. Since protein production has an enormous energy requirement, and mitochondria also plays a vital part in the ER stress response through regulation of energy supply we investigated the possible effect of resveratrol on the bioenergetic status of CHO cells and the possible effect of resveratrol treatment on ER stress.

## 2. Results

**Figure 1:**
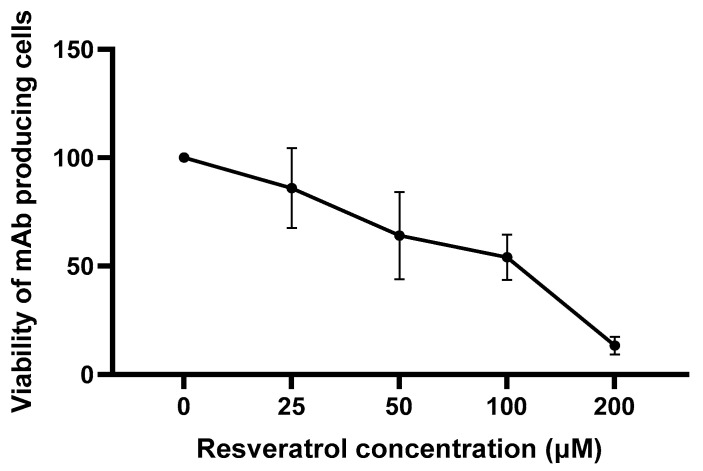
The effect of resveratrol on cell viability. The measured cell count was normalized to each experiment’s own untreated control. mAb-producing CHO cells were treated in 6-well plates for 24 h. Cell viability was determined by flow cytometry as described in the Materials and Methods. Each data point represents the average ± SD from at least three independent experiments.

In the first set of experiments, we found that, similarly to our previous observations [10], resveratrol reduced the viability of the mAb-producing cell line (Figure 1).

**Figure 2:**
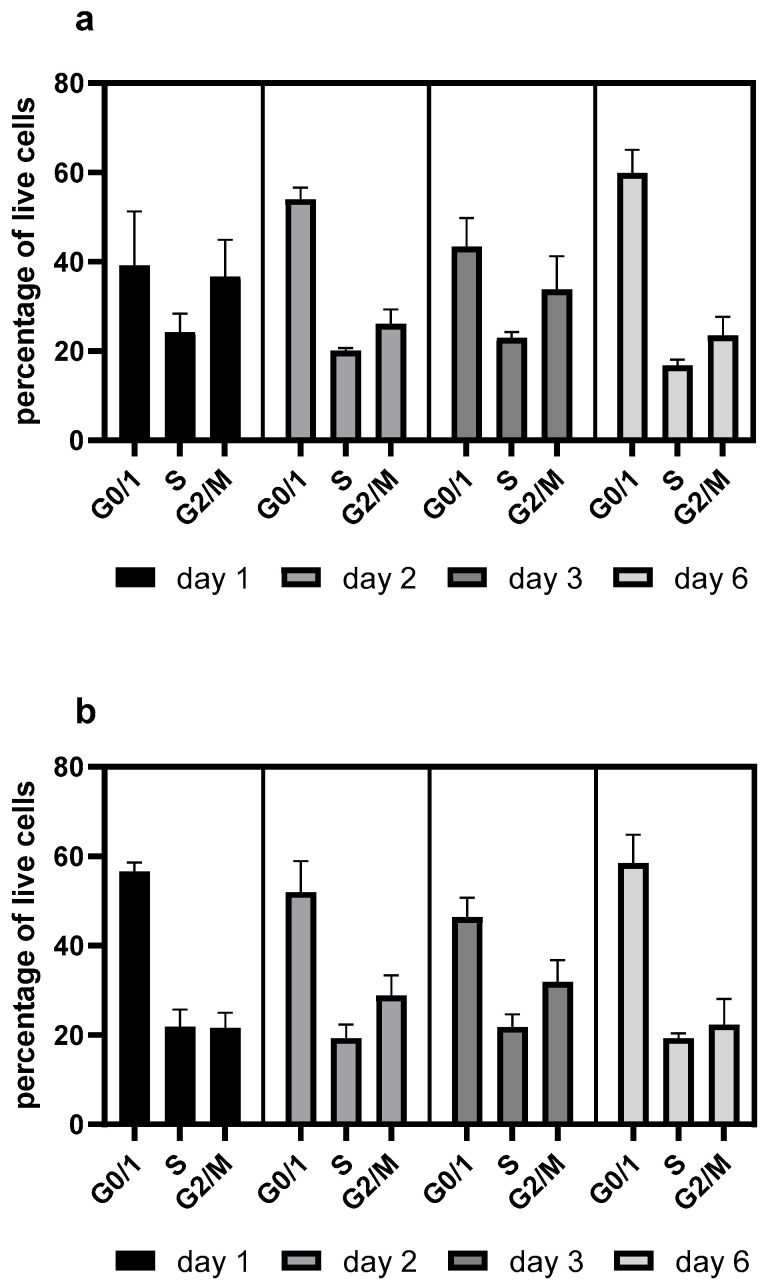
Changes in the cell cycle distribution in mAb-producing CHO cell line following resveratrol treatment. (**a**) Untreated control; (**b**) 50 µM resveratrol treatment. Cell cycle was determined by flow cytometry as described in the Materials and Methods. Cells were categorized into three groups representing the G0/1, S, and G2/M cell cycle phases. CHO cells were grown in 125 mL flasks. Each data point represents the average ± SD from at least three independent experiments.

**Figure 3:**
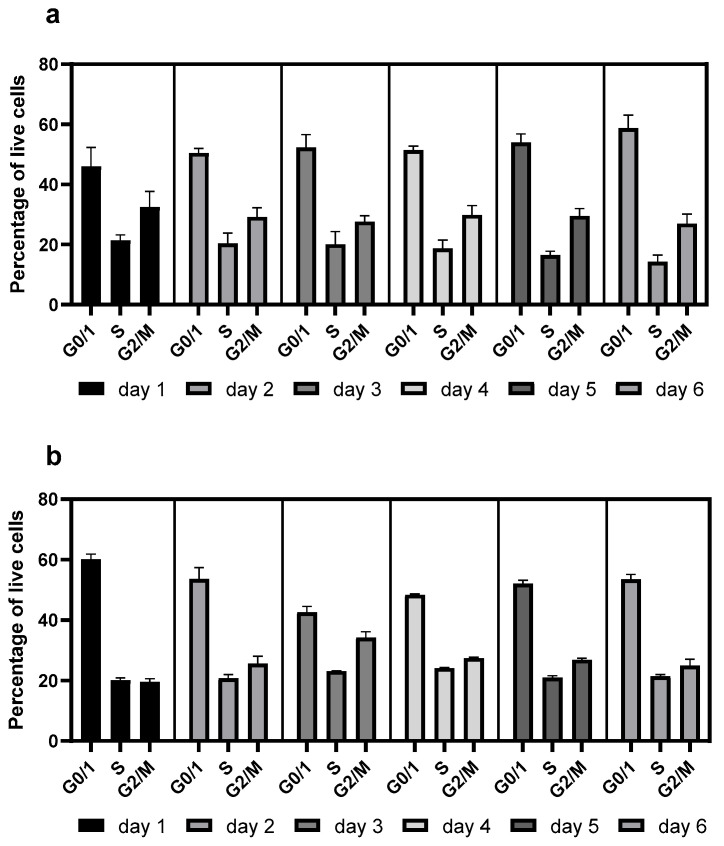
Changes in the cell cycle distribution in mAb-producing CHO cell line following repeated resveratrol treatments. (**a**) Untreated control; (**b**) treated with 50 µM resveratrol at 0 and 72 h. Cell cycle was measured by flow cytometry as described in the Materials and Methods. Cells were categorized into three groups representing the G0/1, S, and G2/M cell cycle phases. CHO cells were grown in 125 mL flasks. Each datapoint represents the average ± SD from at least three independent experiments.

Resveratrol also elevated the proportion of G0/G1-phase cells (Figure 2), consistent with our previous findings [10]. However, this effect was negligible when resveratrol was re-administered after 72 h of seeding (Figure 3).

**Figure 4:**
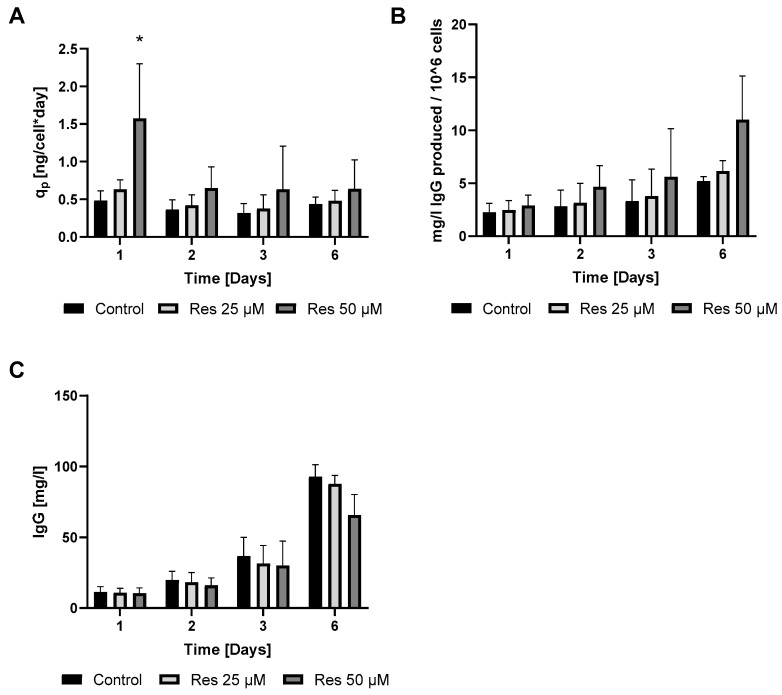
Productivity changes of the mAb-producing cell line following resveratrol treatment. The qp was calculated by dividing the given day’s final protein production by the integral of the VCD curve (**A**). The productivity was also calculated per 10^6^ cells (**B**). IgG concentrations are shown as mg/mL (**C**). Samples were taken every 24 h after the treatment. The cell count was measured with flow cytometry, IgG levels were measured by Cedex^®^ Bio Analyzer as described in the Materials and Methods. Each data point represents the average ± SD from at least three independent experiments. * Significantly different (*p* < 0.05) from the untreated control of the same day.

In perfect concordance with the findings of Toronjo-Urquiza et al. (2019) [4], resveratrol treatment led to a slight decline in IgG synthesis (Figure 4C); however, the calculated productivity increased (Figure 4A,B).

**Figure 5:**
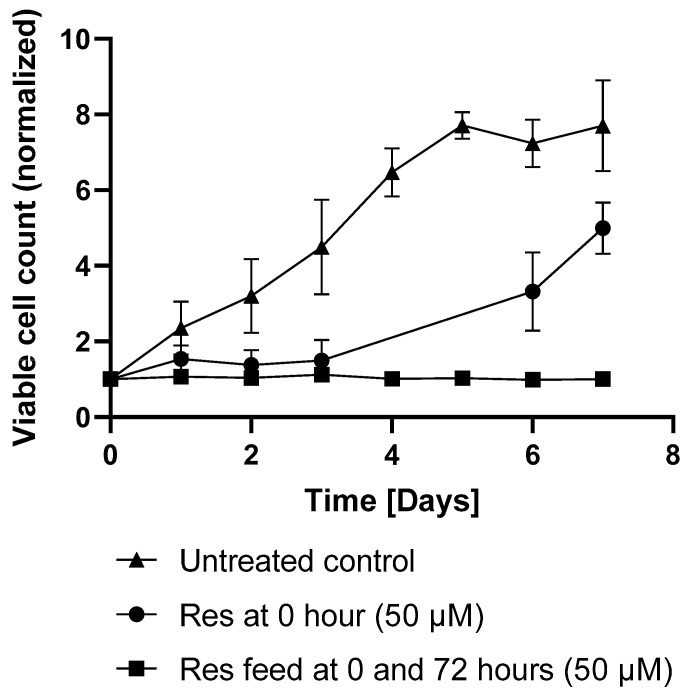
Changes in the growth curve of the mAb-producing cell line following repeated resveratrol treatments. Cell count was normalized to each experiment’s own “day 0” (the initial cell amount). Cell count was measured by flow cytometry as described in the Materials and Methods. Treatment with resveratrol at 0 and 72 h caused a permanently low cell density. Each data point represents the average ± SD from at least three independent experiments.

In the later days of the experiments, the difference in productivity between treated and control batches became negligible. The viable cell counts in the untreated flasks reached a plateau on day 6. A comparable increase in growth was observed in the resveratrol treated flasks after 3 days, corresponding with the decline in resveratrol’s effect (Figure 5).

**Figure 6:**
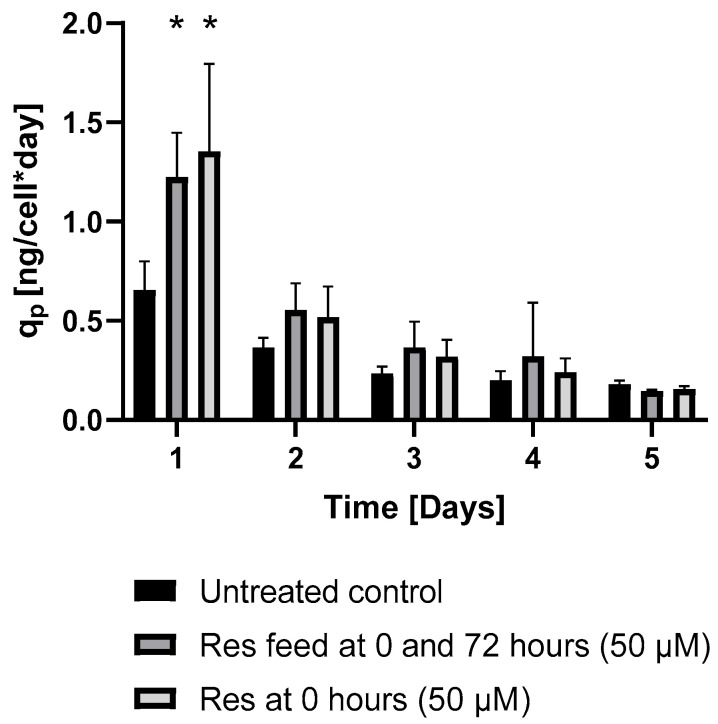
The changes in the productivity of the mAb-producing cell line following repeated resveratrol treatments. The qp was calculated by dividing final protein production of the given day by the integral of the VCD curve. Samples were taken every 24 h after the treatment. Cell count was measured by flow cytometry, IgG levels were measured by Cedex^®^ Bio Analyzer as described in the Materials and Methods. Each data point represents the average ± SD from at least three independent experiments. * Significantly different (*p* < 0.05) from the untreated control of the same day.

**Figure 7:**
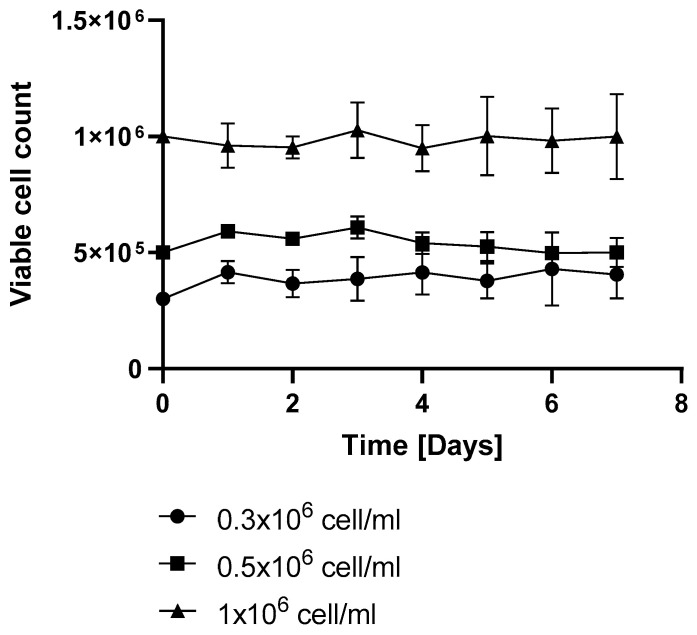
The effect of resveratrol treatment on the viable cell density of mAb-producing cell line. Cell count was measured by flow cytometry as described in the Materials and Methods. Cells were treated with 50 µM resveratrol at 0 and 72 h. Each data point represents the average ± SD from at least three independent experiments.

Resveratrol was administered at the beginning of fermentation and again 72 h after initiation. Resveratrol treatment applied at 72 h resulted in a similar productivity as the initial-treatment-only group or the untreated control (Figure 6). However, repeated supplementation with resveratrol (50 µM; after 72 h) maintained its antiproliferative effect, sustaining the reduced viable cell count (Figure 5). Under repeated resveratrol treatment, the highest VCD of 1.21 +/− 0.1 (normalized to the initial cell count) was measured on day 3 of fermentation (Figure 5). After this point, the initial-treatment-only cells resumed proliferation, while in the flasks receiving repeated resveratrol treatment, the cell number stayed around the initial inoculation cell count, regardless of whether it was 0.3 × 10^6^, 0.5 × 10^6^, or 1 × 10^6^/mL (Figure 7).

**Figure 8:**
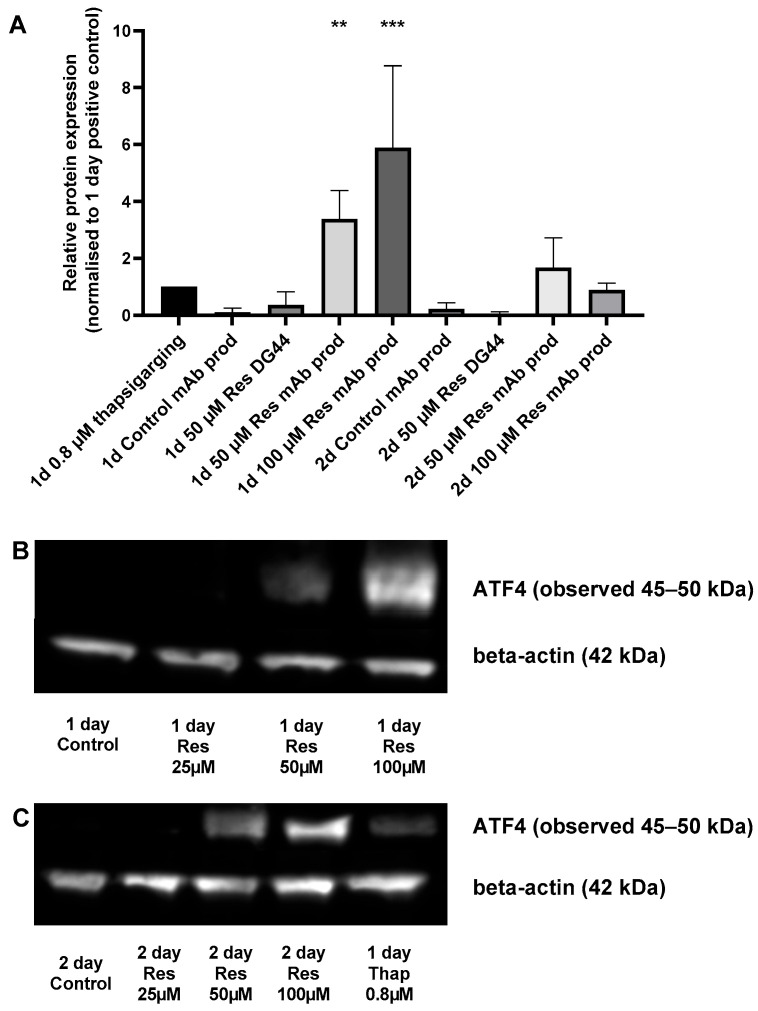
The effect of resveratrol treatment on ATF4 level in mAb-producing CHO cells. Samples were taken at the same time each day. Western blot analysis was performed as described in the Materials and Methods. Beta-actin was used as a loading control, while 0.8 µM thapsigargin was used as a positive control for ER stress. Densitometry data represents the intensity of ATF4 compared to beta-actin. Each data point represents the average ± SD from at least three independent experiments. ** Significantly different (*p* < 0.01); *** (*p* < 0.001) from the same day’s mAb-producing control (**A**). Representative Western blot photos of at least three experiments are shown to demonstrate the change in ATF4 level (**B**,**C**). (Day abbreviated as “d”, resveratrol is abbreviated as “Res”, and mAb-producing as “mAb prod”).

**Figure 9:**
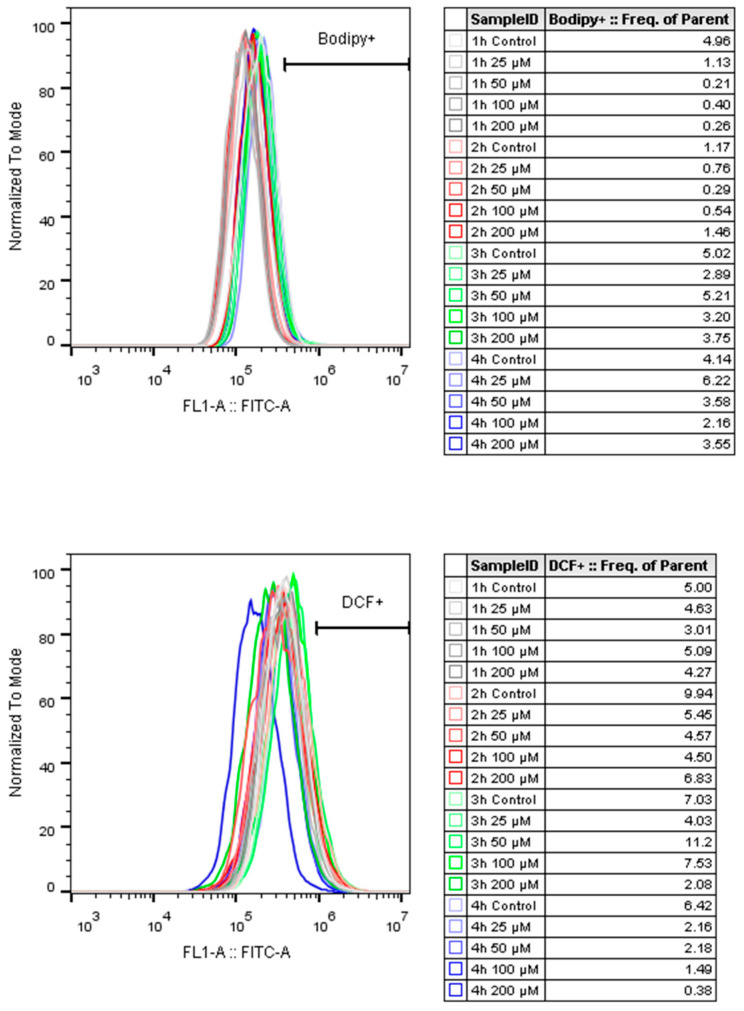
The effect of resveratrol treatment on ROS production in mAb-producing CHO cell line. Lipid peroxidation and ROS levels were measured by flow cytometry as described in the Materials and Methods, using the Bodipy C11 fluorescent probe for lipid peroxidation measurement and the DCF fluorescent probe for ROS measurement. Control samples were fluorescently labeled but untreated. Bodipy+ and DCF+ gates were set to contain 5% of cell in 1 h control samples. One representative is shown from at least three independent experiments. Cell population was gated using SSC-FSC and propidium iodide negative staining for singlet living cells.

ER stress can be triggered by elevated protein production. To assess the potential induction of ER stress, the level of the well-known ER stress marker ATF4 [13] was determined. Resveratrol treatment increased ATF4 expression in a concentration-dependent manner after 24 h (Figure 8). This phenomenon could only be observed in the mAb-producing cells, and not in the non-mAb-producing DG44 line (Figure 8). This difference in ATF4 protein expression can be attributed to the increase observed in mAb production. Furthermore, both the elevation in specific productivity (qp) and ATF4 showed similar temporal patterns (Figure 6 and Figure 8). Interestingly, the observed elevation of ATF4 level was not associated with oxidative stress (Figure 9).

**Figure 10:**
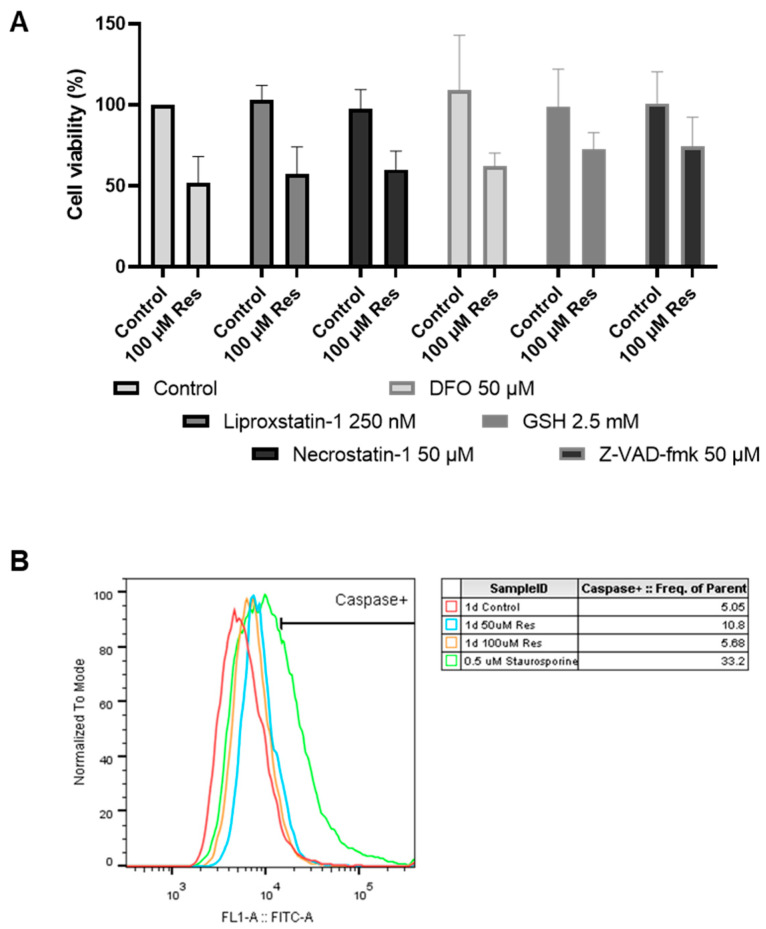
Investigation of the potential cell death types involved in resveratrol-induced cell death. Cell viability was determined by flow cytometry as described in the Materials and Methods (**A**). The potential involvement of apoptosis was also checked by the determination of caspase 3/7 activity (**B**). Cells were seeded and treated on 24-well plates. Both resveratrol and the inhibitors were added directly after seeding. Samples were taken after 24 h. For each experiment, cell count is normalized to untreated cells. Each data point represents the average ± SD from at least three independent experiments.

During the investigation of the potential cell death types, it was found that neither apoptosis, necroptosis, nor ferroptosis played any role in the resveratrol-induced decrease in cell viability (Figure 10).

**Figure 11:**
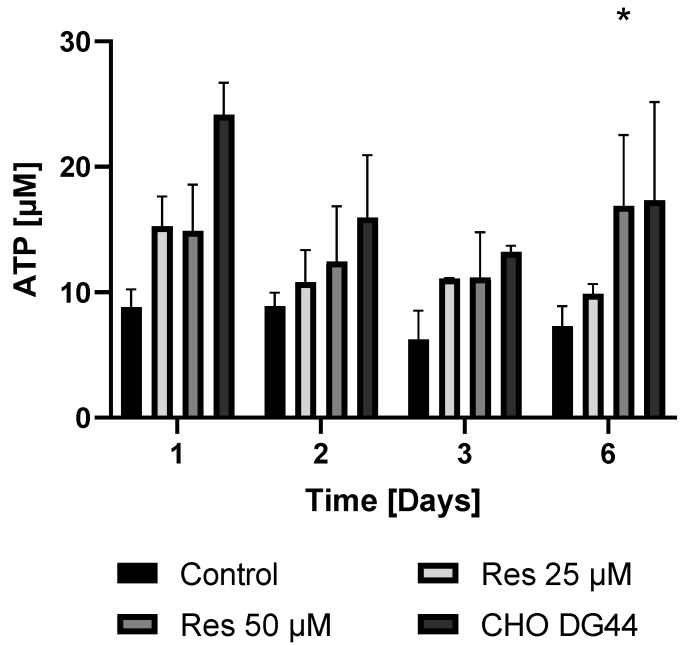
The effect of resveratrol treatment on the cellular ATP levels in mAb-producing and non-producing CHO cell lines. The levels of ATP were measured by HPLC as described in the Materials and Methods. Cells were maintained and treated in 125 mL flasks. Each data point represents the average ± SD from at least three independent experiments. * Significantly different (*p* < 0.05) from the same day’s untreated mAb-producing cell line control.

**Figure 12:**
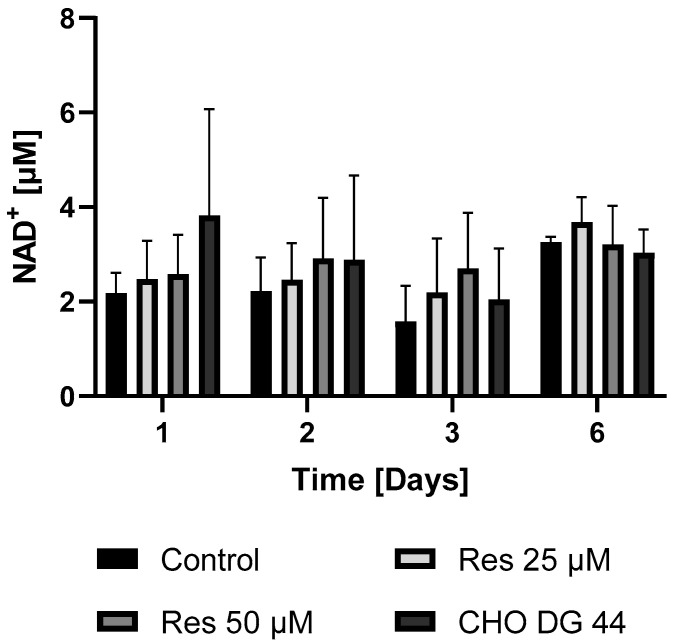
The effect of resveratrol treatment on the cellular NAD^+^ levels in mAb-producing and non-producing CHO cell lines. The levels of NAD^+^ were measured by HPLC as described in the Materials and Methods. Cells were maintained and treated in 125 mL flasks. Each data point represents the average ± SD from at least three independent experiments.

Given that protein synthesis can be associated with extremely high energy demand, it was hypothesized that the enhanced bioenergetics of resveratrol-treated cells may contribute to the elevated mAb productivity. To check this hypothesis, both the ATP and NAD^+^ levels of the resveratrol-treated and non-treated cells were determined in both the mAb-producing cell line and the non-mAb-producing cell line DG44. As expected, the mAb-producing CHO line exhibited lower ATP and NAD^+^ levels compared to the non-mAb-producing DG44 cell line (Figure 11 and Figure 12). Resveratrol treatment resulted in a statistically non-significant increase in both NAD^+^ and ATP levels (Figure 11 and Figure 12). The elevation in the ATP content of the cells only became significant 6 days after 50 µM resveratrol treatment (Figure 11).

**Figure 13:**
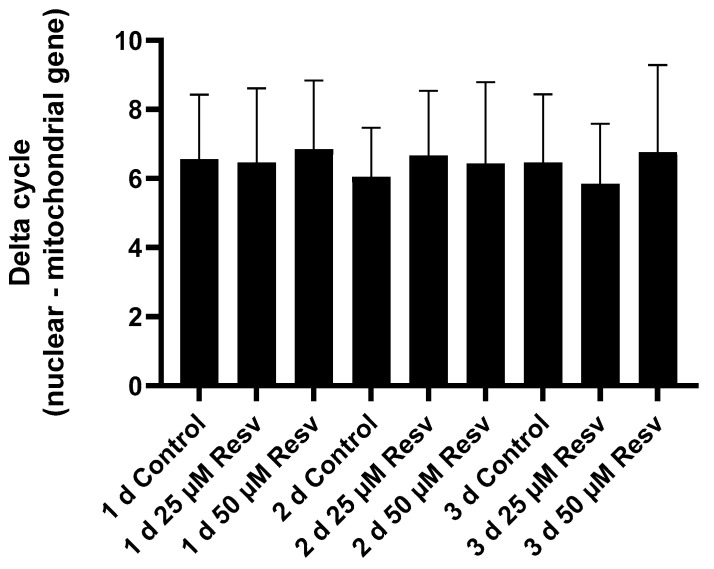
The potential effect of resveratrol on the mtDNA/nDNA ratio. Hemoglobin (HBB) and cytochrome c oxidase (COX) were chosen as representatives for nuclear and mitochondrial DNA, respectively. Their copy number was determined by real-time PCR as described in the Materials and Methods. The average differences between the mitochondrial and nuclear cycles are displayed. Each data point represents the average ± SD from at least three independent experiments.

It was investigated whether the increase in the number of mitochondria could be the cause of the observed increase in ATP levels. However, there was no difference detected in the ratio of mitochondrial to genomic genes between the treated and non-treated cells (Figure 13).

**Figure 14:**
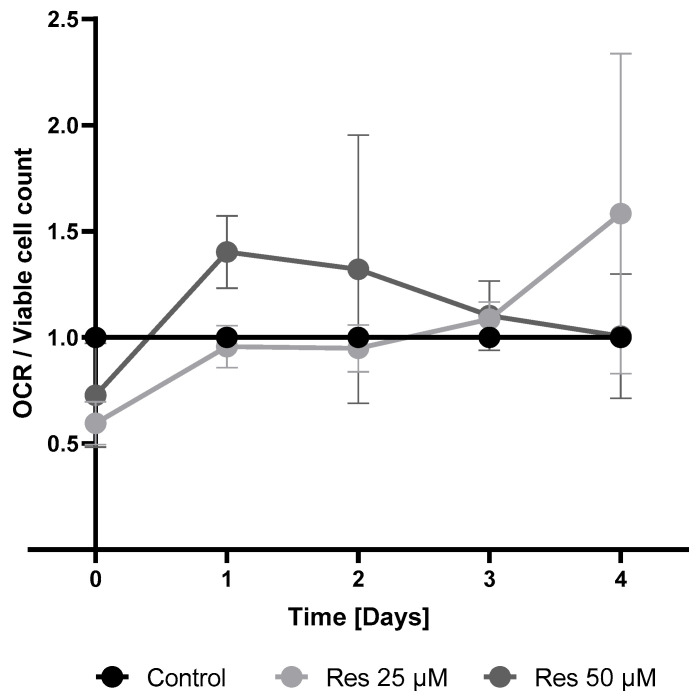
The effect of resveratrol on oxygen consumption rate (OCR)/viable cell count. Each point is compared to the same day’s untreated (control) cells. The OCR value was calculated by the S-nest program, while the viable cell count was measured by flow cytometry as described in the Materials and Methods. The displayed OCR levels were calculated from the 6 OCR values measured before determination of the cell count. Each data point represents the average ± SD from at least three independent experiments.

**Figure 15:**
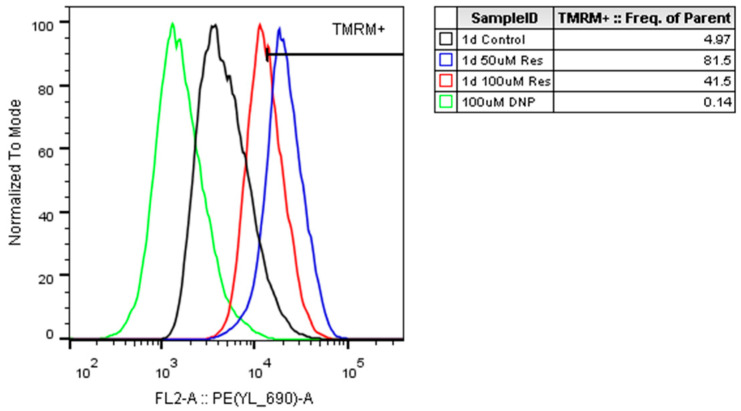
The effect of resveratrol treatment on the mitochondrial membrane potential. Mitochondrial membrane potential was determined by flow cytometry with TMRM (50 nM) as described in the Materials and Methods, where 100 µM of DNP was used as a positive control. Untreated control samples were also fluorescently labeled. The TMRM+ gate was set to contain 5% of cells in the untreated control sample. Cell population was gated using SSC-FSC and propidium iodide negative staining for singlet living cells. One representative experiment is shown from at least three independent experiments.

To investigate the possible acceleration of mitochondrial respiration due to resveratrol treatment, the oxygen consumption rate (OCR) was monitored. Treatment with both 25 µM and 50 µM resveratrol resulted in elevated OCRs after 24 h. However, by the second day the OCR was restored to the control value in the 25 µM treated cells, whereas in 50 µM resveratrol-treated cells the OCR exceeded the control values throughout the entire investigated period (Figure 14)**.** The observed elevation in the mitochondrial membrane potential due to resveratrol treatment (Figure 15) confirmed the elevated OCRs.

## 3. Discussion

The increasing unpredictability of market demands and the growing market of biosimilars both intensify the need to reduce manufacturing costs [14]. Therefore, improving the productivity of CHO cell cultures has been the focus of considerable research and development, leading to the discovery of several different productivity-enhancing strategies. These include improved reactor design, enhanced feed strategies, perfusion systems, modulated temperature conditions, and improved culture media compositions [4,5,14]. Among these, cell culture media additives have significant potential to improve specific productivity [6,15]. A previous study demonstrated that the treatment of CHO cells with resveratrol resulted in a reduction in viable cell density with minimal decrease in IgG synthesis, as well as a significant increase in cell specific productivity [4].

In the present study, we apply our knowledge of resveratrol, gained on immortal cell lines [10,16], to elucidate the details of the effect of resveratrol on mAb-producing CHO cells, with particular attention to the potential ER stress due to the increased productivity induced by resveratrol treatment.

Consistent with previous observations [4,9], resveratrol reduced the viability of the mAb-producing cell line (Figure 1). It should be noted that up to 100 µM concentration, this reduction was moderate and only became pronounced at 200 µM (Figure 1). Resveratrol treatment caused cell cycle arrest, with an elevated proportion of G0/G1-phase cells (Figure 2), which corresponds with previous findings [4,17]. As a result of this cell cycle arrest, IgG synthesis showed a slight decline; however, the productivity of the mAb-producing CHO line was significantly increased on the first day (Figure 4A and Figure 6). A (non-significant) elevation of productivity was maintained until the fourth day (Figure 6). This long-term productivity increase of approximately 20% may seem insignificant at first glance; however, a 20% increase in mAb productivity represents a notable economic benefit since it could be achieved with a cheap and chemically defined additive (resveratrol). Furthermore, if the mAb titer is related to the viable cell number (Figure 4B), the observed increase in mAb titer can contribute to reducing the amount of expensive feed media. It is considered that resveratrol inhibits topoisomerase II by preventing ATPase domain dimerization, which results in cell cycle arrest [18]. Interestingly, resveratrol treatment in a lower concentration (25 μM) induced a decline of the proportion of S-phase cells and a rise in cells in the G1/G0 phase; however, at a higher concentration (50 μM), resveratrol treatment acted inversely and caused a dramatic decline in G1/G0-phase cells and an equally dramatic rise in the S phase [4]. Our observations were similar to the effect of 25 μM treatment, since a significant elevation in the proportion of G0/G1-phase cells was detected after 24 h of resveratrol treatment (Figure 2). In line with previous findings, resveratrol only exerted any effect when administered at the time of inoculation. Its later administration or supplementation after 72 h did not result in any cell cycle arrest or productivity-enhancing effects (Figure 3) [4]. Consequently, mAb expression peaked after 24 h of treatment (Figure 6). The observation that the change in the distribution of cells and the elevation of the productivity was temporary [4] was reinforced by our results (Figure 2 and Figure 6).

The production of a recombinant protein places considerable stress on the cell. On the one hand, the folding of a large amount of protein is likely to trigger ER overload, ER stress, which can often lead to cell death [19]. On the other hand, protein synthesis is one of the most energy-intensive processes, so in many cases the energy supply of the cell can be the limiting factor. Thus, it is no coincidence that slowing down cell proliferation (by resveratrol) increases protein productivity (Figure 6). In the second phase of our experiments, these two factors were investigated.

An excellent marker of ER overload and increased folding pressure is the development of ER stress [20]. Therefore, if ER stress can be detected, there is a good chance that the amount of protein produced by a cell is indeed increased. In our experiments, resveratrol treatment increased the expression of the well-known ER stress marker ATF4 in mAb-producing CHO cells (Figure 8). This ATF4 expression-enhancing effect of resveratrol was absent in the non-mAb-producing CHO line, which suggests that it was indeed triggered by increased mAb production (Figure 8).

Since ER stress can cause depletion of the adaptive capacity of the cell (unfolded protein response), which can ultimately lead to cell death [19,20], we examined whether the most well-known forms of cell death are involved in the resveratrol-induced decrease in cell viability. It was concluded that neither apoptosis, necroptosis, nor ferroptosis was triggered by resveratrol treatment (Figure 10). It was also clarified that oxidative stress did not play a role in the process (Figure 9).

By investigating the bioenergetic background of the cells, we found that due to the high energy demand of protein synthesis, the mAb-producing line exhibited lower ATP levels than the non-mAb-producing line (Figure 11). Treatment of the mAb-producing line with resveratrol clearly increased the ATP content, attenuating the difference between the two lines (Figure 11). A similar trend was observed, although not as pronounced, in cellular NAD^+^ concentration (Figure 12).

There may be several factors behind the increase in ATP levels. A well-known effect of resveratrol is its potential role in mitochondrial biogenesis through the activation of PGC-1α, a master regulator of mitochondrial function. It was proposed that resveratrol could enhance oxidative phosphorylation and ATP production, possibly through SIRT1-dependent deacetylation of PGC-1α, leading to increased mitochondrial density [21]. At the same time, others have found a reduced number of mitochondria due to resveratrol treatment [22].

Therefore, we determined the mitochondrial/nuclear DNA ratio—which correlates well with the number of mitochondria per cell. However, our measurements revealed no change in the number of mitochondria (Figure 13). Based on these results, the hypothesis that resveratrol increased cellular ATP levels through increased mitochondrial biogenesis could be rejected.

Mitochondrial dynamics can be another factor that is also thought to be influenced by resveratrol. It seems resveratrol prevents mitochondrial fragmentation while promoting mitochondrial fusion, resulting in enhanced efficiency of mitochondrial ATP production [23]. Furthermore, resveratrol could enhance mitophagy via the PINK1/Parkin pathway and enhance the clearance of damaged mitochondria [24]. Since we also measured increased oxygen consumption (Figure 14) and an increase in mitochondrial membrane potential (Figure 15) along with unchanged mitochondrial number (Figure 13), we speculated that the enhanced quality of mitochondria due to resveratrol treatment [25] could be in the background of the elevated oxygen consumption and cellular ATP level. Finally, it should play an important role (at least partially) in the observed productivity increase.

In summary, resveratrol induced cell cycle arrest which resulted in increased protein productivity of mAb-producing cells. We demonstrated for the first time that resveratrol induces ER stress in mAb-producing CHO lines, presumably by increasing the amount of mAb produced, without inducing oxidative stress or classical cell death pathways such as apoptosis, necroptosis, or ferroptosis. We report for the first time that resveratrol is able to increase ATP levels in mAb-producing CHO cells, thereby providing additional energy for mAb production.

Necessarily, our results raised novel questions and problems. The ER stress triggered by the resveratrol-induced enhanced protein synthesis poses a substantial bottleneck for production of protein therapeutics [26]. Thus, our future research should focus on the management of this ER stress while maintaining high productivity.

## 4. Materials and Methods

### 4.1. Cell Cultures

Both CHO cell lines were cultured according to ATCC guidelines. Cells were grown in a cell culture incubator at 37 °C, 5% CO_2_, and 100% relative humidity in 125 mL flasks. The chemically defined culture medium used for the mAb-producing cell line was supplemented with glutamine (Thermo Fisher Scientific, Waltham, MA, USA, Gibco™, Waltham, MA, USA, 25030081) to a 4 mM final concentration.

For the DG44 cell line (Thermo Fisher Scientific, CHO DG44 Cells (cGMP banked)) CD-DG44 (1x) (Thermo Fisher Scientific, Gibco™ 12610010), culture medium was supplemented with 0.1% Anti-Clumping Agent (Thermo Fisher Scientific, Gibco™, 0010057AE) and Poloxamer 188 Non-ionic Surfactant (100X) (Thermo Fisher Scientific, Gibco™, 24040032). Both cell lines were subcultured routinely every 3 or 4 days, with an initial seed concentration of 0.3  ×  10^6^ viable cells/mL.

### 4.2. Cell Treatments

For experiments requiring multiple samplings over an extended period, cells were subcultured at a density of 0.3  ×  10^6^ viable cells/mL into new 125 mL flasks and were supplemented with various compounds. Sampling was performed under sterile conditions every 24 h.

For 24 h long treatments, 24-well plates were used. Each well contained 1 mL of cell suspension with a concentration of 0.3  ×  10^6^ viable cells/mL and was then treated with various compounds.

To measure the cytotoxicity of resveratrol (MedChemExpress, Princeton, NJ, USA, HY-16561), a concentration range from 25 to 200 µM was applied.

To investigate the mechanism of resveratrol-induced cell death, 100 µM (based on our measurements, in this concentration, resveratrol induces approximately 50% cell lethality) was used in combination with different compounds that have known programmed cell death inhibiting effects. The final concentrations of the inhibitors were as follows: 250 nM Liproxstatin-1 (MCE^®^, HY-12726, dissolved in DMSO), 50 µM Necrostatin-1 (Santa Cruz Biotechnology, Dallas, TX, USA, sc-200142, dissolved in DMSO), 50 µM Deferoxamine (Sigma-Aldrich^®^, St. Louis, MO, USA, D9533), 2.5 mM GSH (Sigma-Aldrich^®^, G4251), 50 µM Z-VAD-FMK (MCE^®^, HY-16658B, dissolved in DMSO). These concentrations were used based on our previous experiments [27].

Protein samples were collected from 125 mL flasks with a one-time treatment of various resveratrol concentrations. In addition, 0.8 µM Thapsigargin (Sigma-Aldrich, T9033) was used as a positive control for ER stress, selected from a concentration range of 0.2 to 2 µM based on prior measurements.

### 4.3. Measurement of Cell Viability with Attune Flow Cytometer

All cell viability measurements were performed on the Attune NxT Flow Cytometer (Thermo Fisher Scientific). In total, 50 µL of a 150 µL cell suspension sample was measured each time. The viable cells were gated based on SSC-FSC and propidium iodide negative staining prior to these experiments, separately for both cell lines.

### 4.4. Measurement of ROS and Lipid Peroxidation Using Flow Cytometry

Cells were treated in 24-well plates, 0.3  ×  10^6^ viable cells/mL/well. For measurement, at chosen times, 6 wells of cells were harvested, pooled, and washed two times with PBS (centrifuged at 500× *g* for 3 min), then resuspended in HBSS (Hank’s Balanced Salt Solution, Sigma-Aldrich^®^). Cells were equally distributed onto a 96-well plate, with a final concentration of 5 µM Dichlorofluorescein-diacetate (DCF-DA, Thermo Fisher Scientific™, D399) or 2 µM BODIPY™ 581/591 C11 (Bodipy C11, Thermo Fisher Scientific™, D3861) for flow cytometry. The dyes were used separately to avoid interference. After the plate was incubated for 30 min at 37 °C, the cells were analyzed with a CytoFLEX™ S (Beckman Coulter™, Brea, CA, USA) Flow Cytometer. The cell populations were gated using unstained cells. The data were analyzed using FlowJo^®^ (Becton, Dickinson and Company; version: 10.0.7r2). Histograms are normalized to the sample’s mode.

### 4.5. Isolation and Quantitation of Protein Samples

Cells were sampled from 125 mL flasks every 24 h after treatment. Cells were lysed in RIPA protein isolation buffer (150 mM NaCl, 1% NP-40, and 50 mM Tris pH 8.0) supplemented with 1% protease inhibitor cocktail (Sigma-Aldrich^®^, P8340), 1% phosphatase inhibitor cocktail (Sigma-Aldrich^®^, P0001), and 1 mM PMSF (Thermo Fisher Scientific™, 36978). Samples were incubated on ice for 30 min, then centrifuged at 14,000× *g* for 15 min at 4 °C. The supernatant was stored at −80 °C until analysis.

Protein samples were quantified using the Pierce™ BCA Protein Assay Kit (Thermo Fisher Scientific™, 23225), according to the manufacturer’s guidelines.

### 4.6. Analysis of Protein Samples Using Western Blot

SDS-PAGE was carried out using a Mini Gel Tank (Thermo Fisher Scientific™, Invitrogen™ Waltham, MA, USA, A25977), with pre-packaged gels (NuPAGE™ Bis-Tris Mini Protein Gels, 4–12%, 1.0–1.5 mm, NP0336BOX). Proteins were transferred onto Millipore 0.45 µM nitrocellulose membranes with using the Power Blotter System (Thermo Scientific™, PB0013) and the Power Blotter 1-Step™ Transfer Buffer (Thermo Scientific™, PB7100). Immunoblotting was performed using TBS-Tween (0.1%) containing 5% non-fat dry milk as a blocking solution and 1% non-fat dry milk for antibody solutions. Loading was controlled by developing β-Actin (Proteintech, 20536-1-AP) on the membrane in each experiment. The anti-activating transcription factor 4 (anti-ATF4; Proteintech, 10835-1-AP) antibody was used as an endoplasmic reticulum stress indicator. As a secondary antibody, HRP-Goat Anti-Rabbit IgG (Proteintech^®^, Rosemont, IL, USA, 00001-2) was used.

The bands were visualized using Clarity™ ECL Western Blotting Substrate chemiluminescence detection kit (Bio-Rad, Hercules, CA, USA, 170-5060) and the VWR™ (Radnor, PA, USA) Imager Chemi Premium gel documentation system with VWR™ Image Capture Software (version: 1.6.1.0). Densitometry analysis was carried out using ImageJ (version: 1.53k) software bundled with 64-bit Java 1.8.0_172.

### 4.7. Isolation of DNA Samples

Cell were grown in 125 mL flasks, cell concentrations were determined by flow cytometry; then, 0.1  ×  10^6^ cells were used for DNA isolation. The cells were washed with PBS two times (centrifuged at 500× *g* for 3 min), then placed into Nuclei Lysis Solution for 15 min. DNA isolation were performed with the Wizard^®^ SV Genomic DNA Purification System (Promega, Madison, WI, USA, A2360), according to the manufacturer’s guidelines. The samples were stored at −80 °C until analysis.

### 4.8. Real-Time PCR

The PCR mix was assembled based on previous experiments according to the following. For each well on the PCR plate, 10 µL of SybR Green master mix (KAPA SYBR^®^ FAST, Merck, SFUKB Roche, KK4600), 5 µL of the sample, 3.4 µL nuclease-free water (Thermo Fisher Scientific, AM9914G), and 0.8 µL of forward and reverse primers (10 µM) were used. Two pairs of primers were used: HBB gene primers for the nuclear DNA (Hemoglobin subunit beta; fw: 5′-GTTGTCATTTCCTATTTCTCCAGCA; rev: 5′-AGCAACCCTATTGCCCTAGC) and COX-2 primers for the mitochondrial DNA measurements (Cyclooxygenase-2; fw: 5′-GGCTTACCCATCTCAATTAGGC; rev: 5′-ACTGCTGGCAAGATAGTTCAAATG). To run the experiments, a PikoReal 96 Real-Time PCR System (Thermo Fisher Scientific™, TCR0096) was used, set up with the PikoReal Software 2.2 (Thermo Fisher Scientific™). Each experiment ran over 40 cycles, with an annealing temperature of 60 °C. Cq results were saved and used for later calculations.

### 4.9. Preparing ATP and NAD^+^ Samples

Cells were treated in 125 mL flasks and sampled every 24 h. Each sample contained 0.1  ×  10^6^ viable cells. The cells were washed with PBS two times (centrifuged at 500× *g* for 3 min). The supernatant was discarded, and the cells were resuspended in 150 μL 5% sulfosalicylic acid (SSA), then incubated on ice. After 15 min, the cells were centrifuged at 21,200× *g* for 5 min at 4 °C. The supernatant was neutralized with 50 μL 3 M potassium hydrogen phosphate solution and stored at −80 °C until analysis.

### 4.10. Measurement of ATP and NAD^+^ with UPLC

NAD^+^ analysis was based on the method from Yoshino J. and Imai S. [28]. For separation, a Waters Acquity UPLC H-Class system (Waters, Milford, MA, USA) was used, equipped with an Acquity UPLC BEH C18 2.1 × 50 mm column with an average particle diameter of 1.7 μm. Gradient elution was used with 50 mM potassium hydrogen phosphate (pH 7.0) and methanol. The detector was a Waters Acquity PDA detector; absorbance was monitored at 261 nm. Quantitation was achieved by measuring NAD^+^ standards (Reanal Laboratory Chemicals Ltd., Budapest, Hungary, 53-84-9).

ATP analysis was based on a method from Juarez-Facio A. T. et al. [29]. For separation, a Waters Acquity UPLC H-Class system (Waters, Milford, MA, USA) was used, equipped with an OOB-4760-YO Luna^®^ Omega Polar C18 100 Å 50 × 3.0 mm column (Phenomenex^®^, Torrance, CA, USA) with an average particle diameter of 3 μm, using isocratic elution with a mobile phase consisting of 50 mM potassium hydrogen phosphate (pH 6.80). The detector was a Waters Acquity PDA detector; absorbance was monitored at 254 nm. Quantitation was achieved by measuring ATP standards (Sigma-Aldrich^®^, A2383).

### 4.11. Measurement of IgG

The samples were taken from 125 mL flasks and centrifuged at 1000× *g* for 3 min. The supernatant was pipetted into the sample holders of the Cedex^®^ Bio Analyzer (Roche Diagnostics, Indianapolis, IN, USA). Each further step was performed according to the manufacturer’s guidelines. Multiple kits were used throughout the experiments, all manufactured by Roche Diagnostics: Cleaner Alkaline Bio (06689485001), NH_3_ Bio (06343775001), NaCl Diluent 9% Bio (06684700001), LDH Bio (06343767001), IgG Bio (06681743001), Glucose Bio (06343732001), Glutamine V2 Bio (07395655001), Glutamate V2 Bio (07395582001). Productivity was calculated from the results of IgG measurements by dividing final protein production by the integral of the VCD curve, based on Toronjo-Urquiza L et al. [9].

### 4.12. Cell Cycle Analysis

To measure the cell cycle distribution, a staining solution (Thermo Fischer Scientific, FxCycle™ PI/RNase Staining Solution, F10797) was used. In total, 0.1  ×  10^6^ cells were sampled and washed with PBS two times (centrifuged at 500× *g* for 3 min). The cells were resuspended in 70% EtOH and placed on ice for 15 min, then centrifuged at 1000× *g* for 3 min. Finally, 150 µL of the FxCycle solution was added to each sample, and measurement with flow cytometry was performed after 15 min incubation at room temperature in the dark.

### 4.13. Oxygen Consumption Rate

A total of 1.6 mL medium with 0.3  ×  10^6^ cells/mL was seeded into each well of the microbioreactor plate (S.NEST™, CYTENA, Freiburg, GER). Cells were treated with resveratrol at final concentrations of 0, 25, or 50 μM. DO (dissolved oxygen) data were collected by the instrument at 10 min intervals. Viable cells were counted every day with flow cytometry. Mixing was at maximum, with 30 sec/cycle. The cells were kept at 37 °C, 5% CO_2_, and 100% relative humidity.

### 4.14. Measurement of Mitochondrial Membrane Potential and Caspase Activity

Cells were maintained in 125 mL flasks, treated at inoculation, and sampled at 24, 48, and 72 h. Suspensions containing 0.2  ×  10^6^ cells were centrifuged at 500× *g* for 3 min, then resuspended in 200 µL culture medium.

To measure mitochondrial membrane potential, a final concentration of 50 nM of TMRM (Invitrogen™, T668) fluorescent dye was added to the samples, and the cells were incubated for 30 min at 37 °C. For the positive control, 100 µM DNP (Sigma-Aldrich^®^, D198501) was added to 0.2  ×  10^6^ cells 10 min before staining.

To measure caspase activity, a final concentration of 5 µM CellEvent™ fluorescent dye (Thermo Fisher Scientific, Invitrogen™ CellEvent™ Caspase-3/7 Detection Reagents, C10423) was added to the samples, and the cells were incubated for 20 min in the dark, at room temperature. As a positive control, cells were treated with a final concentration of 0.5 µM Staurosporine (Sigma-Aldrich^®^, S4400) for 4 h before staining.

In both cases, after incubation, the samples were centrifuged at 500× *g* for 3 min, the supernatant was discarded, and the cells were resuspended in 200 µL PBS. Then, cells were measured with a CytoFLEX™ S (Beckman Coulter™, Brea, CA, USA) Flow Cytometer. The cell populations were focused with unstained cells. The data were analyzed using FlowJo^®^ software (Ashland, OR: Becton, Dickinson and Company; version: 10.0.7r2). Histograms are normalized to the sample’s mode.

### 4.15. Statistical Analyses

GraphPad Prism 9 version 9.5.1 (Dotmatics) was used for statistical analysis and data visualization. *p* < 0.05 compared to the same-day (if applicable) untreated control was considered statistically significant, marked by an asterisk. For determining significance, ordinary one-way and two-way ANOVA were used.

## Data Availability

The data that support the findings of this study are available from the corresponding author upon reasonable request.
